# Validation of a novel numerical model to predict regionalized blood flow in the coronary arteries

**DOI:** 10.1093/ehjdh/ztac077

**Published:** 2023-01-03

**Authors:** Daniel J Taylor, Jeroen Feher, Krzysztof Czechowicz, Ian Halliday, D R Hose, Rebecca Gosling, Louise Aubiniere-Robb, Marcel van’t Veer, Danielle C J Keulards, Pim Tonino, Michel Rochette, Julian P Gunn, Paul D Morris

**Affiliations:** Department of Infection, Immunity and Cardiovascular Science, University of Sheffield, Sheffield, UK; ANSYS Research and Development, Lyon, France; Department of Infection, Immunity and Cardiovascular Science, University of Sheffield, Sheffield, UK; Department of Infection, Immunity and Cardiovascular Science, University of Sheffield, Sheffield, UK; Insigneo Institute for In Silico Medicine, Sheffield, UK; Department of Infection, Immunity and Cardiovascular Science, University of Sheffield, Sheffield, UK; Insigneo Institute for In Silico Medicine, Sheffield, UK; Department of Infection, Immunity and Cardiovascular Science, University of Sheffield, Sheffield, UK; Insigneo Institute for In Silico Medicine, Sheffield, UK; Department of Cardiology, Sheffield Teaching Hospitals NHS Foundation Trust, Sheffield, UK; Department of Infection, Immunity and Cardiovascular Science, University of Sheffield, Sheffield, UK; Department of Cardiology, Catharina Hospital, Eindhoven, Netherlands; Department of Biomechanical Engineering, Eindhoven University of Technology, Eindhoven, Netherlands; Department of Cardiology, Catharina Hospital, Eindhoven, Netherlands; Department of Cardiology, Catharina Hospital, Eindhoven, Netherlands; Department of Biomechanical Engineering, Eindhoven University of Technology, Eindhoven, Netherlands; ANSYS Research and Development, Lyon, France; Department of Infection, Immunity and Cardiovascular Science, University of Sheffield, Sheffield, UK; Insigneo Institute for In Silico Medicine, Sheffield, UK; Department of Cardiology, Sheffield Teaching Hospitals NHS Foundation Trust, Sheffield, UK; Department of Infection, Immunity and Cardiovascular Science, University of Sheffield, Sheffield, UK; Insigneo Institute for In Silico Medicine, Sheffield, UK; Department of Cardiology, Sheffield Teaching Hospitals NHS Foundation Trust, Sheffield, UK

**Keywords:** Coronary flow, Bifurcation, Computational Fluid Dynamics

## Abstract

**Aims:**

Ischaemic heart disease results from insufficient coronary blood flow. Direct measurement of absolute flow (mL/min) is feasible, but has not entered routine clinical practice in most catheterization laboratories. Interventional cardiologists, therefore, rely on surrogate markers of flow. Recently, we described a computational fluid dynamics (CFD) method for predicting flow that differentiates inlet, side branch, and outlet flows during angiography. In the current study, we evaluate a new method that regionalizes flow along the length of the artery.

**Methods and results:**

Three-dimensional coronary anatomy was reconstructed from angiograms from 20 patients with chronic coronary syndrome. All flows were computed using CFD by applying the pressure gradient to the reconstructed geometry. Side branch flow was modelled as a porous wall boundary. Side branch flow magnitude was based on morphometric scaling laws with two models: a homogeneous model with flow loss along the entire arterial length; and a regionalized model with flow proportional to local taper. Flow results were validated against invasive measurements of flow by continuous infusion thermodilution (Coroventis™, Abbott). Both methods quantified flow relative to the invasive measures: homogeneous (*r* 0.47, *P* 0.006; zero bias; 95% CI −168 to +168 mL/min); regionalized method (*r* 0.43, *P* 0.013; zero bias; 95% CI −175 to +175 mL/min).

**Conclusion:**

During angiography and pressure wire assessment, coronary flow can now be regionalized and differentiated at the inlet, outlet, and side branches. The effect of epicardial disease on agreement suggests the model may be best targeted at cases with a stenosis close to side branches.

## Graphical Abstract

**Graphical Abstract:**
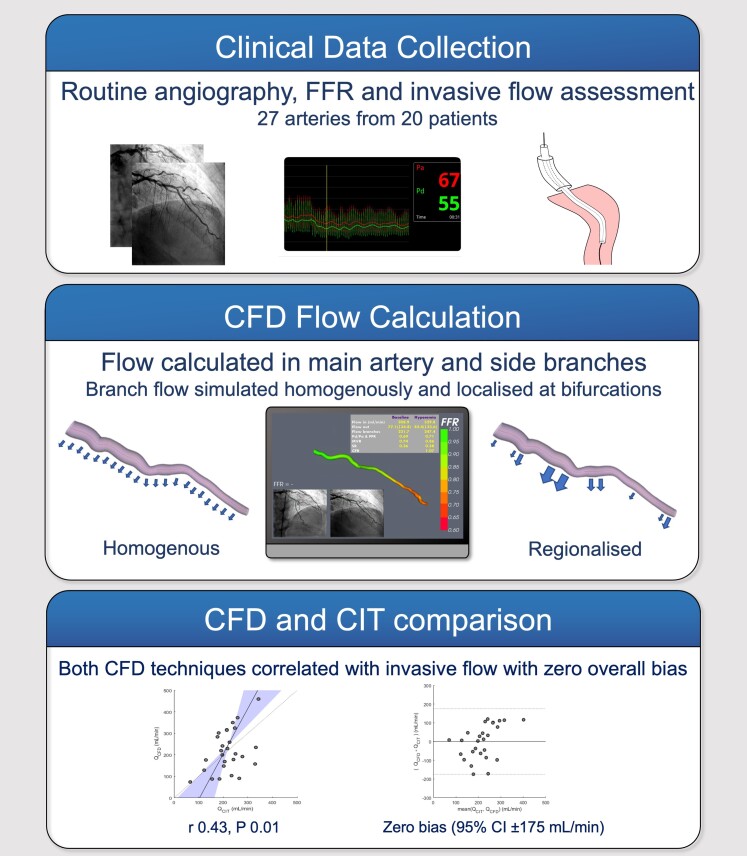

## Introduction

Ischaemic heart disease (IHD) is the leading cause of death worldwide. IHD results from an insufficiency of coronary blood flow (Q), commonly caused by occlusive coronary arterial disease and encompasses a wide variety of clinical syndromes including symptomatic ischaemia (angina), myocardial infarction, and heart failure. Interventional treatments are effective in restoring Q, but should only be targeted at lesions that result in ischaemia. There is, however, no technique available for routine clinical use in the cardiac catheterization laboratory which directly measures Q. Over several decades, cardiologists have relied upon indirect surrogate markers, such as thermodilution-derived mean transit time, Doppler-derived flow velocity, and pressure-derived fractional flow reserve (FFR) and related indices^1–3^ and, more recently, ‘virtual’ FFR (vFFR), computed from angiographic images.^4^ All these methods have strengths and weaknesses, but none measure ‘absolute’ Q in mL/min. FFR is the ratio of distal to proximal translesional pressure measurements, but it only expresses *fractional* and not absolute reductions in Q and it cannot diagnose coronary microvascular disease (MVD); a common but often overlooked cause of IHD.^5–10^

Two methods have been developed to quantify Q. The first, continuous infusion thermodilution (CIT), is the most established and validated method and uses the Rayflow™ infusion catheter (Hexacath, Paris, Fr).^11,12^ The second, virtuQ™, derives Q from a computational fluid dynamics (CFD) simulation based upon the 3D angiographic anatomy and pressure wire measurements, and is the subject of this study.^13^ Both methods also quantify absolute microvascular resistance (Rmicro) and so provide a comprehensive evaluation of the entire coronary circulation. A limitation of the CFD method was that it only considered the main vessel and was agnostic to side branch flow.^13^ While this may be acceptable for computing vFFR, which simply interrogates the effect of a localized lesion, it is less so for computing Q, which is dependent upon the lesion, side branches, and microvasculature.^14^ In a recent study, we described a porous wall simulation method that used established morphometric scaling laws and case-specific knowledge of arterial taper to allow blood to virtually ‘leak’ out of the main vessel proportional to side branch flow.^14^ A limitation of this approach was that side branch flow was distributed diffusely across the entire length of the main vessel. This approach, therefore, failed to capture the haemodynamic effects of large volumes of regionalized flow loss to major arterial side branches.

In this study, we describe a novel method that attempts to correlate side branch flow ‘leak’ to areas of significant bifurcation and therefore represents the pattern of flow commonly encountered in real coronary arteries. We aimed to validate this new method against CIT measurements and compare results with those from the homogeneous CFD method.

## Methods

CFD analyses were performed at the University of Sheffield, UK on retrospective clinical data collected from adult patients (≥18 years old) with suspected myocardial ischaemia with non-obstructive coronary arteries (INOCA) undergoing coronary angiography and Rayflow catheterization at the Catharina Hospital, Eindhoven, NL. Patients provided informed consent and the study was approved by the research ethics board [Medical research Ethics Committees United (MEC-U)]. All supporting data are provided in this manuscript and in the *supplementary material*. A derivation analysis of Murray’s exponent, based upon the same raw clinical dataset as this study, has already been published.^15^

### Clinical data collection

FFR was assessed in arteries of interest using standard technique (PressureWire™ X, Abbott, MN, USA), with a maximum tolerated signal drift of 2 mmHg,^16^ and the Rayflow™ infusion catheter (Hexacath, Paris, Fr) and the Coroventis™ (Abbott, Plymouth, MN) system,^11,12^ were used to quantify Q (Q_CIT_) and Rmicro (Rmicro_CIT_). Thermodilution measurements were taken in the proximal coronary artery, with pressure readings under saline-induced hyperaemia taken at the location of 6 cm distal to the tip of the infusion catheter.^12^ Pseudonymized angiography (DICOM) and physiological (pressure and flow) data were exported to the University of Sheffield for computational processing in February 2021. Full exclusion criteria have previously been published.^15^ In brief, cases were excluded if transferred clinical data were unsuitable for computational processing or major arterial side branches were present within 3.0 cm of the Rayflow™ infusion port. The latter criterion was used because side branches close to the infusion port can draw a significant portion of saline infusate and therefore affect Q_CIT_ and Rmicro_CIT_ results.^11^ A recommended correction for the partial luminal occlusion created by the infusion catheter was applied (Supplementary material online, *S1*). Visual percentage stenosis was evaluated by two interventional cardiologists and quantitative coronary angiography (QCA) was used to grade 2D- and 3D-percentage stenosis.

### Simulating coronary blood flow

A complete description of the virtuQ method and arterial reconstruction process has been published previously.^13,17^ Two angiographic projections of the vessel of interest, acquired ≥30° apart and during end-diastole, were used to reconstruct coronary anatomy. Image selection and a correction for table movement between angiographic runs was manually performed, while centreline tracing and vessel border detection was performed semi-automatically from contrast gradient for both images with manual correction if required. Finally, a rigid, 3D, axisymmetric geometry representative of patient anatomy was automatically created. The inlet of reconstructed arteries corresponded to the location of invasive Q_CIT_ and *P_a_* measurement, while the outlet corresponded to the location of *P_d_*. Invasive pressure readings were used to define inlet and outlet boundary conditions. The CFD simulation was executed using standard blood parameters (density 1056 kg/m^3^; viscosity 0.0035 Pa s), modelling steady, laminar flow of a Newtonian fluid. The suitability of these assumptions has been demonstrated previously.^18–20^

### Simulating side branch flow

In this study, side branch flow was simulated by modelling arterial reconstructions with a porous wall boundary. This allowed flow loss from the main vessel lumen. For all circumstances, inlet flow = outlet flow + side branch flow. Side branch magnitude was inferred from taper of the main vessel using Murray’s law,^21^ which relates the diameters (*D*) of parent vessel (PV) and daughter branches (DB) around a bifurcation:


$$D_{\mathrm{PV}}^{x}=\;D_{\mathrm{DB}1}^{x}+\;D_{\mathrm{DB}2}^{x}$$

**Figure 1:**
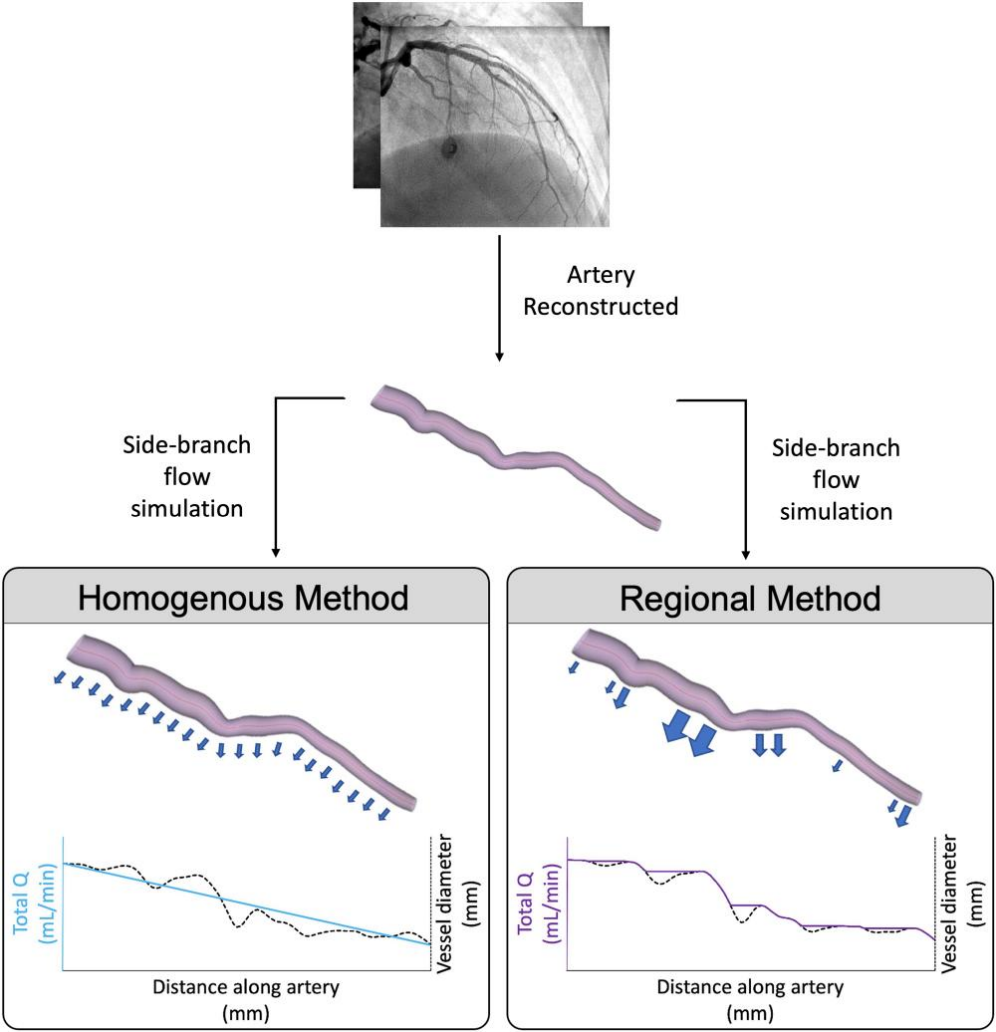
Schematic illustrating different leak profiles for homogeneous and regional methods, blue arrows represent side branch losses. When using the homogeneous method, Q throughout the reconstructed artery is agnostic to local perturbations in vessel radius. When using the regional method, Q is dependent upon local vessel radius, while no flow is leaked at points of downstream radius recovery: indicating a stenosis and not healthy taper.

In which ‘*x*’ represents a constant whose value varies between 2.0 and 3.0.^22–24^ The magnitude of side branch flow (i.e. flow across the porous wall) was dependent upon the value used for this proportionality constant (which was optimized in a previous study^15^) and the method used to distribute side branch losses (homogeneous vs. regional, see below). The aim of this study was to compare accuracy of the homogeneous and regional techniques against the invasively derived measures. *The homogeneous porous wall method* distributed side branch losses consistently along the entire length of the reconstructed vessel and was unaffected by local taper. *The regional porous wall method* distributed side branch flow loss proportionally to the local healthy vessel taper. This was done to better regionalize Q. In healthy vessels, regional taper occurs at branch points. Thus, this method should more accurately regionalize blood flow in the *in-vivo* artery. To distinguish healthy vessel taper from diameter reduction caused by atherosclerotic plaque, a stenosis detection filter was used which excluded porous wall leak in sections with downstream recovery of vessel diameter (*Figure 1*). It was therefore imperative that the inlet and outlet of reconstructed vessels were selected in healthy sections of vessel. All reconstructions were processed blind to the intra-coronary pressure and CIT results.

### Intra-observer variability

To assess variability in physiological results that may be introduced by subtle differences in reconstructed coronary anatomy during the manual phases of artery reconstruction, intra-operator variability was assessed for CFD-derived Q (QCFD) and Rmicro (RmicroCFD) across three repeated reconstructions of the five most and least stenosed cases.

### Statistical analysis

Categorical variables are presented as frequency (percentage). Normally distributed continuous variables are presented as mean (standard deviation), while those with a non-normal distribution are presented as median [interquartile range]. Normality of data distribution was assessed using the Shapiro–Wilk test. For parametric data, mean values of haemodynamic parameters were compared using an unpaired *t*-test, while a Mann–Whitney *U* test was used for non-parametric data. Correlation was quantified using Pearson’s correlation coefficient (*r*) and agreement was assessed with both Passing and Bablok regression and Bland Altman plots. Log-transformation was used for skewed datasets. Analyses were performed using MATLAB R2021a (Math Works Inc., USA).

## Results

### Clinical cases

From the original 48 cases, 27 cases from 20 patients yielded full physiological datasets. Cases were excluded due to insufficient angiographic quality (*n* = 2) and pressure (*n* = 1) data, insufficient angiographic projections (*n* = 11), unsuitable patient anatomy (*n* = 3), coronary intervention performed prior to Q_CIT_ measurement (*n* = 2), and major side branches present ≤3.0 cm of the Rayflow catheter (*n* = 2). Seven patients (35%) were male, the mean age was 62 (±10) years, and the mean body mass index (BMI) was 25.2 (±3.6) kg/m^2^. The 27 cases included left anterior descending (LAD) arteries (*n* = 18), left circumflex (Cx) arteries (*n* = 7), and right coronary arteries (RCA) (*n* = 2). The mean Q_CIT_ was 219 (±61) mL/min and the median Rmicro_CIT_ was 360 [290–450] mmHg min/L. The majority of included cases contained minimal epicardial disease, with a mean FFR of 0.87 (±0.08) and only three cases meeting a clinical threshold of FFR significance (≤0.80). Median percentage stenosis assessed by operator, 2D QCA and 3D QCA was 10% [0–25%], 16% [0–31%], and 15% [0–33%], respectively (full details of cases shown in *Table 1*). No included cases contained diffuse epicardial disease. Using a threshold of 460 mmHg min/L,^25–27^ five (25%) patients had clinically significant MVD as assessed by Rmicro_CIT_.

**Table 1:** Reconstructed vessel characteristics and flows

Case		FFR	Percentage stenosis			Q results (mL/min)		Rmicro results (mmHg min/L)	
Vessel	Number		Operator	2D	3D	Q_CIT_	Q_CFD_	Rmicro_CIT_	Rmicro_CFD_
LAD	01	0.87	10	16	11	343	459	250	184
	02	0.82	0	15	22	182	302	340	191
	03	0.69	10	29	34	192	219	320	276
	04	0.83	15	31	30	197	241	320	259
	05	0.83	15	9	8	215	316	310	196
	06	0.85	5	17	16	202	148	410	547
	07	0.84	5	19	11	177	283	280	164
	08	0.81	0	0	0	278	192	210	274
	09	0.86	20	16	15	217	229	350	330
	10	0.86	0	0	0	330	157	210	410
	11	0.97	0	0	0	234	103	360	761
	12	0.74	35	43	55	66	74	1140	992
	13	0.83	40	31	33	123	129	520	462
	14	0.95	25	18	37	185	88	460	969
	15	0.93	0	0	0	239	350	320	203
	16	0.81	10	10	11	226	258	390	329
	17	0.91	35	33	32	155	87	450	793
	18	0.93	20	32	39	206	169	400	482
LCx	19	0.98	0	0	0	333	234	290	402
	20	0.95	45	53	43	247	324	360	253
	21	0.88	50	39	33	215	314	600	392
	22	0.78	40	31	15	130	176	580	420
	23	0.93	15	9	12	259	373	270	176
	24	0.96	0	0	0	197	199	540	521
	25	0.96	0	0	0	265	91	250	716
RCA	26	0.96	5	12	19	242	178	400	538
	27	0.97	10	26	23	250	204	390	465

All reported CFD results acquired using the regional porous wall boundary method.

Q_CIT_, absolute coronary flow measured with Rayflow catheter after correction applied (mL/min); Rmicro_CIT_, microvascular resistance measured with Rayflow catheter after correction applied (mmHg min/L); QCA, quantitative coronary angiography; LAD, left anterior descending artery; LCx, circumflex artery; RCA, right coronary artery.

### The homogeneous porous wall boundary method

**Figure 2:**
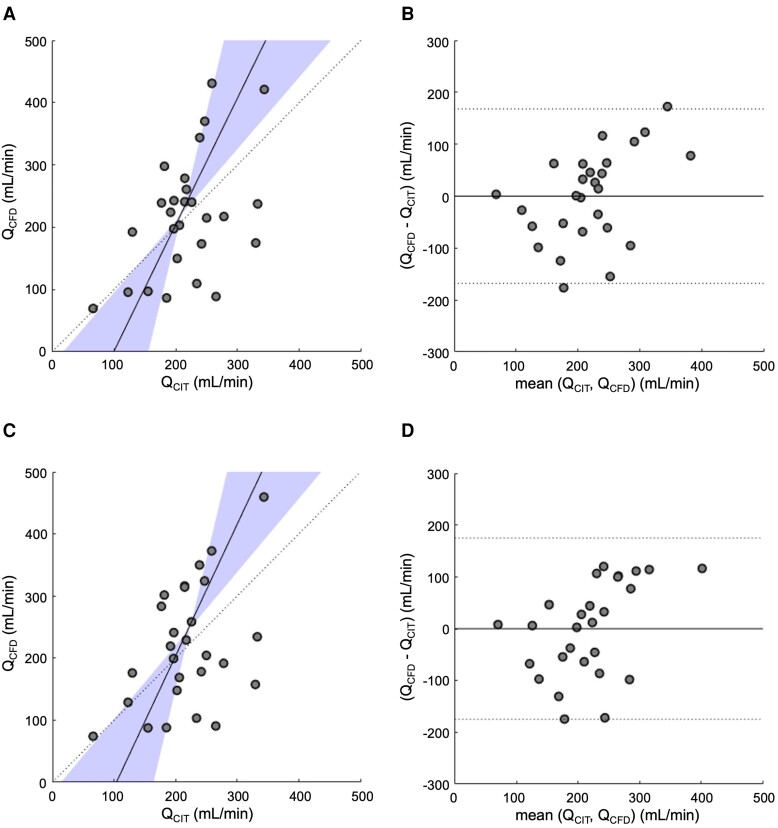
Correlation and agreement between Q_CFD_ and Q_CIT_. (*A*) Passing and Bablok showing correlation between Q_CFD_ and Q_CIT_ for the homogeneous porous wall boundary method. (*B*) Bland Altman plot showing agreement between Q_CFD_ and Q_CIT_ for the homogeneous porous wall boundary method. (*C*) Passing and Bablok showing correlation between Q_CFD_ and Q_CIT_ for the regional porous wall boundary method. (*D*) Bland Altman plot showing agreement between Q_CFD_ and Q_CIT_ for the regional porous wall boundary method.

**Figure 3:**
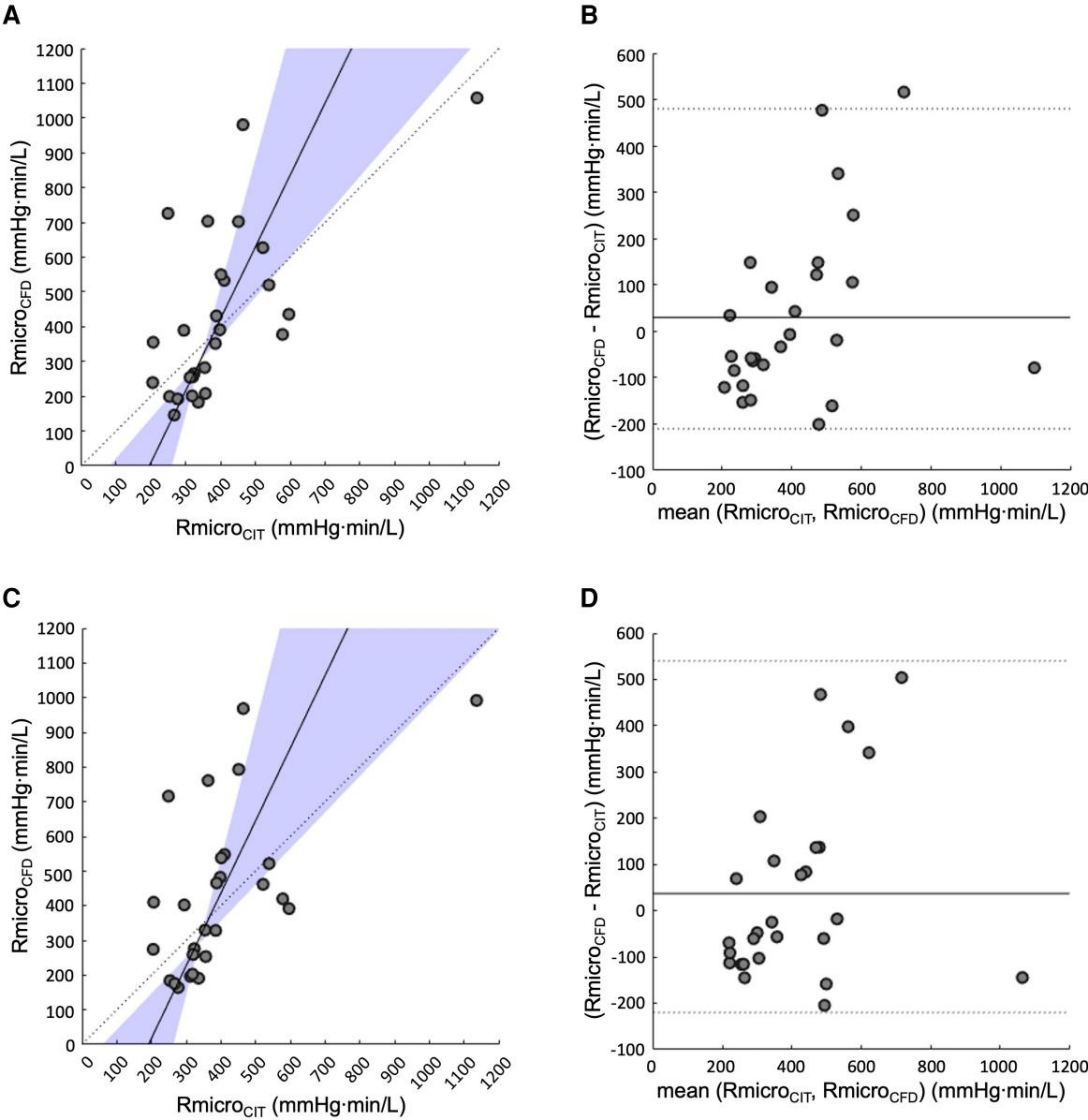
Correlation and agreement between Rmicro_CFD_ and Rmicro_CIT_. (*A*) Passing and Bablok showing correlation between Rmicro_CFD_ and Rmicro_CIT_ for the homogeneous porous wall boundary method. (*B*) Bland Altman plot showing agreement between Rmicro_CFD_ and Rmicro_CIT_ for the homogeneous porous wall boundary method. (*C*) Passing and Bablok showing correlation between Rmicro_CFD_ and Rmicro_CIT_ for the regional porous wall boundary method. (*D*) Bland Altman plot showing agreement between Rmicro_CFD_ and Rmicro_CIT_ for the regional porous wall boundary method.

The homogeneous porous wall boundary method disclosed a mean Q_CFD_ of 219 (±86) mL/min. There was a statistically significant correlation between Q_CFD_ and Q_CIT_ (*r* 0.473, *P* 0.006), Passing and Bablok regression identified constant and proportional differences between techniques (*c* coefficient −202, 95% CI −633 to −20; *m* coefficient 2.03, 95% CI 1.15 to 4.07), the mean delta between techniques was zero and the 95% Bland Altman limits of agreement were −168 to +168 mL/min (*Figure 2*). The proportional differences between Q_CFD_ and Q_CIT_ was characterized by an increase in bias for higher flow rates, which is visually displayed by Bland Altman analysis. A significant correlation between Rmicro_CFD_ and Rmicro_CIT_ was also observed (*r* 0.647, *P* 0.0001), constant and proportional differences were present (*c* coefficient −400, 95% CI −950 to −90; *m* coefficient 2.07, 95% CI 1.15 to 3.67), the mean delta between techniques was +30 mmHg min/L and the 95% Bland Altman limits of agreement were −210 to +480 mmHg min/L (*Figure 3*).

### The regional porous wall boundary method

The regional porous wall boundary method disclosed a mean Q_CFD_ of 219 (±96) mL/min. The correlation between Q_CFD_ and Q_CIT_ was significant (*r* 0.429, *P* 0.0127), Passing and Bablok regression identified constant and proportional differences between techniques (*c* coefficient −220, 95% CI −687 to −16; *m* coefficient 2.12, 95% CI 1.18 to 4.19), the mean delta between techniques was zero and the 95% Bland Altman limits of agreement were −175 to +175 mL/min (*Figure 2*). Agreement between Q_CFD_ and Q_CIT_ was not superior for either the homogeneous or regional techniques (*t* 0.0023, *P* 0.998). A significant correlation between Rmicro_CFD_ and Rmicro_CIT_ was also observed (*r* 0.586, *P* 0.0006), constant and proportional differences were present (*c* coefficient −400, 95% CI −1030 to −60; *m* coefficient 2.09, 95% CI 1.04 to 3.90), the mean delta between techniques was +37 mmHg min/L and the 95% Bland Altman limits of agreement were −220 to +540 mmHg min/L (*Figure 3*). Agreement between Rmicro_CFD_ and Rmicro_CIT_ was not superior for either the homogeneous or regional techniques (U 363, *P* 0.944) (full CFD results shown in *Table 1*). Using a threshold of 460 mmHg min/L, seven (35%) patients had clinically significant MVD as assessed by Rmicro_CFD_. Using Rmicro_CIT_ as gold standard measurements, the sensitivity, specificity, positive predictive value, and negative predictive value of the CFD technique was 80%, 80%, 57%, and 92%, respectively.

### Determinants of agreement

Certain patient and vessel characteristics did appear to influence agreement between the CFD and invasive measurements. For homogeneous and regional porous wall boundary methods, respectively, Q agreement was significantly correlated with translesional pressure drop (*P_a_* − *P_d_*) (*r* 0.449, *P* 0.0094; *r* 0.391, *P* 0.0217), FFR (*r* −0.399, *P* 0.0196; *r* −0.334, *P* 0.0441) and percentage stenosis assessed by 2D (*r* 0.355, *P* 0.0345; *r* 0.472, *P* 0.0065) and 3D QCA (*r* 0.369, *P* 0.0292; *r* 0.489, *P* 0.0048). This meant, for both homogeneous and regional techniques, that agreement between Q_CFD_ and Q_CIT_ improved in cases with greater disease burden assessed by pressure wire studies, 2D QCA and 3D QCA. For the regional method, Q agreement also correlated with visually assessed stenosis (*r* 0.371, *P* 0.0282).

For Rmicro, both homogeneous and regional techniques correlated with translesional pressure drop (*r* 0.359, *P* 0.0330; *r* 0.340, *P* 0.0415) and FFR (*r* −0.368, *P* 0.0295; *r* −0.364, *P* 0.0310), but no effect was seen for any assessment of stenosis (full results are shown in *Table 2*). This meant, for both homogeneous and regional techniques, that agreement between Rmicro_CFD_ and Rmicro_CIT_ improved in cases with greater disease burden assessed by pressure wire studies, but disease burden assessed by percentage stenosis was not associated with agreement.

**Table 2:** Correlations between Q_CFD_ and Q_CIT_ agreement and Rmicro_CFD_ and Rmicro_CIT_ agreement with various arterial reconstruction and demographic variables

	Homogeneous method		Regional method	
	Pearson’s *r*	*P*	Pearson’s *r*	*P*
**Q**				
Percentage stenosis				
ȃVisual assessment	0.237	0.117	0.371	0.0282
ȃ2D QCA	0.355	0.0345	0.471	0.0065
ȃ3D QCA	0.369	0.0292	0.489	0.0048
Pressure wire assessment				
ȃ*P_a_* − *P_d_*	0.449	0.0094	0.391	0.0217
ȃFFR	−0.399	0.0196	−0.334	0.0441
Vessel taper				
ȃInlet diameter − outlet diameter	−0.313	0.0561	−0.194	0.166
**[Rmicro]**				
Percentage stenosis				
ȃVisual assessment	−0.020	0.462	0.028	0.445
ȃ2D QCA	0.172	0.195	0.184	0.179
ȃ3D QCA	0.064	0.375	0.066	0.372
Pressure wire assessment				
ȃ*P_a_* − *P_d_*	0.359	0.0330	0.340	0.0415
ȃFFR	−0.368	0.0295	−0.364	0.0310
Vessel taper				
ȃInlet diameter − outlet diameter	0.136	0.249	0.132	0.256

QCA, quantitative coronary angiography; *P_a_*, proximal pressure under adenosine-induced hyperaemia (mmHg); *P_d_*, distal pressure under adenosine-induced hyperaemia (mmHg).

### Intra-operator variability

For the homogeneous and regional porous wall boundary methods, Q_CFD_ variability was 7.8% (±4.2%) and 5.6% (±2.7%), respectively. For Rmicro_CFD_, this was 2.6% [4.6–6.1%] and 3.2% [1.9–10.2%], respectively. Intra-observer variability did not differ between homogeneous and regional techniques for either Q_CFD_ (U 33, *P* 0.218) or Rmicro_CFD_ (U 45, *P* 0.739).

## Discussion

In this retrospective study, we have validated a regionalized porous wall boundary method for simulating side branch Q and compared the results with those from the original homogeneous method.^15^ The main aim of this work was to regionalize the side branch and main branch flow. This was achieved with no major difference in overall side branch flow compared with the homogeneous method. The new regionalized method correlated with CIT measurements, with zero bias and 95% limits of agreement of ±175 mL/min. Agreement with invasive clinical measures was suboptimal, which we believe resulted from inclusion of several INOCA cases which have minimal stenosis and pressure gradient.

### Current indices for estimating flow

The superiority of translesional pressure indices over standard angiography alone is well documented^9,10^ and has led to the emergence of FFR as the current ‘gold standard’ evaluation of an epicardial lesion’s haemodynamic significance. However, this technique expresses *percentage* reduction in flow and does not quantify MVD. Combined use of FFR with other indices of coronary flow, such as coronary flow reserve (CFR) and index of microvascular resistance (IMR), allows for quantification of disease in both epicardial and microvascular compartments, but this requires additional time and hardware and is currently reserved for a relatively small number of patients. Additionally, the binary nature of treatment thresholds means different indices may conflict and all fail to quantify a direct determinant of myocardial ischaemia – absolute Q. The CIT technique addresses these issues but requires a dedicated infusion catheter and accuracy may be decreased in arteries with multiple, large bifurcations. In contrast, virtuQ may quantify Q at the inlet, outlet, and side branches of reconstructed arteries, along with all of the above-mentioned indices of flow from standard angiography and pressure wire assessment.

### How this technique compares with others

The Rayflow CIT and virtuQ CFD methods quantify Q within the cardiac catheterization laboratory. Previously, one direct *in-vitro* validation of virtuQ outlet Q results has been performed, reporting a bias of +2.08 ± 3.45 mL/min^13^ and our previous validation of inlet Q_CFD_ using the homogeneous porous wall boundary method reported zero bias between CIT results with 95% limits of agreement of ±168 mL/min.^15^ More data are available for the latest, monorail Rayflow catheter design. An *in-vitro* trial of Q_CIT_ reported a bias of −6.5 ± 15.5 mL/min^12^ and one animal study reported a bias of +5 ± 8 mL/min from Q measured in 12 pigs (mean Q 37 mL/min).^28^ A direct validation study of the CIT method with [^15^O]H_2_O positron emission tomography (PET) in 25 patients referred for coronary angiography showed a bias between techniques of −0.9 ± 35 mL/min (95% limits of agreement −70 to +68 mL/min; mean Q across all included vessels 176 mL/min).^29^

In the present study, we reported moderate correlation between CIT and CFD Q for the regional porous wall boundary method. The 95% limits of agreement were ±175 mL/min. These limits of agreement are larger than reported in previous studies of different datasets,^12,13,28,29^ even when accounting for the larger mean Q of patients included in the current study. Several factors may have negatively influenced agreement, the most important of which is likely to be characteristics of included arteries. Q_CFD_ accuracy is critically dependent upon agreement between simulated flow patterns and those occurring *in-vivo*. In healthy arteries, coronary flow is governed predominantly by Poiseuille (viscous) effects and as such, results are extremely sensitive to errors in reconstruction diameter. To put this into context, in a theoretical case taking the average outlet diameter and Q_CFD_ of cases included in this study, an error in outlet reconstruction diameter of ± one single pixel would vary outlet Q_CFD_ from −42 to +62 mL/min (assuming Poiseuille’s law with a constant pressure gradient across the artery). This error will be further magnified for inlet Q_CFD_, when side branch flow is accounted for. Conversely, the complex flow patterns observed in diseased arteries reduce the dominance of Poiseuille effects on flow local to the disease, thereby reducing the sensitivity of Q_CFD_ to small errors in reconstruction diameter. This phenomenon is evidenced by both the correlations observed between Q_CFD_ and Q_CIT_ agreement with percentage luminal stenosis and translesional pressure gradients; both of which are markers of disease severity, and the clustering of cases without any appreciable epicardial disease at the higher error ranges. This means agreement between the two methods would likely have improved through inclusion of cases with moderate to severe epicardial disease.

Furthermore, the CIT method is dependent upon assumptions such as the complete mixing between saline infusate and blood prior to side branches and negligible heat loss to the vessel wall. These conditions may not be observed in patients, therefore resulting in inaccuracies of the CIT technique.^12,28,29^ It is therefore important to consider the limits of agreement reported in this study as an amalgamation of the errors of both CFD and CIT methods and not solely attribute them to either technique.

The absence of any significant difference in *total* side branch flow between the homogeneous and regional porous wall boundary method was reassuring. The main advantage of the regionalized technique is that it seeks to concentrate side branch flow to the location of side branches. This may have advantages for more precise flow predictions when planning intervention just proximal or distal to significant bifurcation points. This, however, is more challenging to validate given that there are no methods that accurately predict coronary blood flow with this level of localization.

Intra-operator variability was excellent for both Q_CFD_ and Rmicro_CFD_. For both porous wall boundary methods, intra-observer variability was less than 5% for outlet Q_CFD_ and 4% for Rmicro_CFD_, which is considerably lower than previously reported results, of 10% and 11% for the same parameters, respectively.^13^

### Limitations

The number of included patients was modest and case exclusion rate was high, but this in keeping with similar retrospective computational studies and no exclusion criteria were applied on successfully modelled cases to improve accuracy. A disproportionate number of LAD arteries were included in this study. Moreover, our model of side branch flow relies on vessel taper, and the proximal RCA has less taper due to a relative lack of major side branches. For both these reasons, the model may lack generalisability for RCA modelling. Future studies will therefore aim to include more RCA’s and include posterior descending and posterior left ventricular branches. Of the included arterial cases, only two (7%) met the criteria for moderate stenosis (>40%) as graded by either 2D or 3D QCA and three (11%) met the FFR threshold of haemodynamic significance (≤0.80). As previously described, this has negative implications for the accuracy of Q_CFD_ and provides the first clinical, supporting evidence of the previously reported requirement for a translesional pressure drop.^13^ Because this was a retrospective analysis of angiograms captured at another centre, the protocols were not optimized for virtuQ processing^4^ which may have affected reconstruction accuracy of included cases. Also, the correction calculation to accommodate for the partial coronary arterial lumen occlusion introduced by the *in-situ* Rayflow catheter is currently unvalidated. Clinically, the patient cohort for which absolute Q assessment will be of value and thresholds for intervention are currently unknown and subject to ongoing research.^30^ Fournier et al. have previously reported a difference in hyperaemic LAD flow between healthy and mildly disease arteries of 65 mL/min.^27^ Given the reported error in the current study, at the current stage of development, virtuQ assessment of absolute Q in minimally diseased arteries may be of limited clinical utility. Furthermore, the porous wall boundary method assumes the inlet and outlet of reconstructed arteries are healthy, so the tool may not be applicable for patients with diffuse epicardial disease. Finally, while this CFD technique quantifies epicardial arterial Q, it cannot assess myocardial flow and may therefore be misleading in the presence of a major collateral blood supply.

## Conclusion

Using a CFD model of coronary arterial blood flow, Q can be quantified and regionalized within the epicardial coronary arteries with flow loss from the main lumen concentrated at the location of side branches. This retrospective study showed no overall difference in performance between the regional and homogeneous techniques. Although the cases met the basic criteria for modelling, the lack of stenosis and relatively small pressure gradient negatively influenced results compared with the invasive technique. Further validation work is required, but this approach may be useful in predicting local blood flow dynamics, particularly in cases with a stenosis close to significant side branches.

## Supplementary Material

ztac077_Supplementary_Data
