# Supplementary material for: Validation of a novel numerical model to predict regionalized blood flow in the coronary arteries
Source: Eur Heart J Digit Health. 2023 Jan 3;4(2):81–9. doi: 10.1093/ehjdh/ztac077 (PMC10039427; doi:10.1093/ehjdh/ztac077)
Supplement: ztac077_Supplementary_Data [file ztac077_supplementary_data.docx]

**Supplementary material**

**S1. Rayflow correction calculation**

Because the Rayflow™ catheter itself reduces the effective diameter of the artery, a correction must be applied to predict what Q would be without the catheter in situ (Q_thermc_). Where FFR_adenosine_ is the FFR measured under adenosine-induced maximal hyperaemia with the pressure-wire (without the Rayflow™ catheter) and FFR_saline_ is the FFR measured under saline-induced maximal hyperaemia with the Rayflow™ catheter in the coronary artery, Q_thermc_ was calculated as follows.

$$\text{Q}_{\text{thermc}}\text{ = }\frac{\text{Q}_{\text{therm}}}{\text{1 - (}\text{FFR}_{\text{adenosine }}\text{- }\text{FFR}_{\text{saline}}\text{)}}$$
